# Acute and Chronic Effects of COVID-19 on the Cardiovascular System

**DOI:** 10.3390/jcdd8100128

**Published:** 2021-10-05

**Authors:** Victor Arévalos, Luis Ortega-Paz, Juan José Rodríguez-Arias, Margarita Calvo López, Leticia Castrillo-Golvano, Anthony Salazar-Rodríguez, Marta Sabaté-Tormos, Francesco Spione, Manel Sabaté, Salvatore Brugaletta

**Affiliations:** 1 Department of Cardiology, Clinic Cardiovascular Institute, Hospital Clinic, 08036 Barcelona, Spain; varevalos88@gmail.com (V.A.); lgortega@clinic.cat (L.O.-P.); juanjose.rodriguez.a@gmail.com (J.J.R.-A.); mcalvol@clinic.cat (M.C.L.); castrillo@clinic.cat (L.C.-G.); aesalazar@clinic.cat (A.S.-R.); francescospio@gmail.com (F.S.); masabate@clinic.cat (M.S.); 2 Institut d’Investigacions Biomèdiques August Pi i Sunyer (IDIBAPS), University of Barcelona, 08036 Barcelona, Spain; 3 Department of Medicine, Medical School, Universitat de Barcelona, 08036 Barcelona, Spain; martasabate1@hotmail.com

**Keywords:** coronavirus disease 2019, SARS-CoV-2, myocardial injury, myocarditis, myocardial infarction, pulmonary embolism, long-term outcome

## Abstract

COVID-19 has shown significant morbidity with the involvement of multiple systems, including the cardiovascular system. Cardiovascular manifestations in the acute phase can include myocardial injury itself, myocardial infarction, venous thromboembolic events, myocarditis, Takotsubo syndrome, and different arrhythmic events. Myocardial injury defined by the rise of cardiac biomarkers in blood has been found in multiple studies with a prevalence of about 20%. Its presence is related to worse clinical outcomes and in-hospital mortality. The mechanisms of myocardial injury have been the subject of intense research but still need to be clarified. The characterization of the cardiac affectation with echocardiography and cardiac magnetic resonance has found mixed results in different studies, with a striking incidence of imaging criteria for myocarditis. Regarding post-acute and chronic follow-up results, the persistence of symptoms and imaging changes in recovered COVID-19 patients has raised concerns about the duration and the possible significance of these findings. Even though the knowledge about this disease has increased incredibly in the last year, many aspects are still unclear and warrant further research.

## 1. Introduction

Coronavirus disease 2019 (COVID-19) has become a community health issue, causing social and economic problems in almost all countries worldwide. The pandemic has overloaded the health systems due to its fast-spreading and high morbidity in some populations, reaching high mortality rates even in developed countries such as the U.S., where COVID-19 was the third leading cause of death during 2020 [1,2].

COVID-19 is caused by the severe acute respiratory syndrome coronavirus-2 (SARS-CoV-2). The pathophysiologic mechanism of this virus was mainly related to an acute respiratory distress syndrome (ARDS) and systemic severe inflammatory reaction [3]. One of the first observations at the beginning of the pandemic was that patients with comorbidities, including previous cardiovascular disease, were more likely to present a worse clinical outcome, including a higher risk of death [4,5]. Along with the pandemic’s evolution, several reports informed about the potential multisystemic and cardiac involvement related to COVID-19 [6].

**Figure 1:**
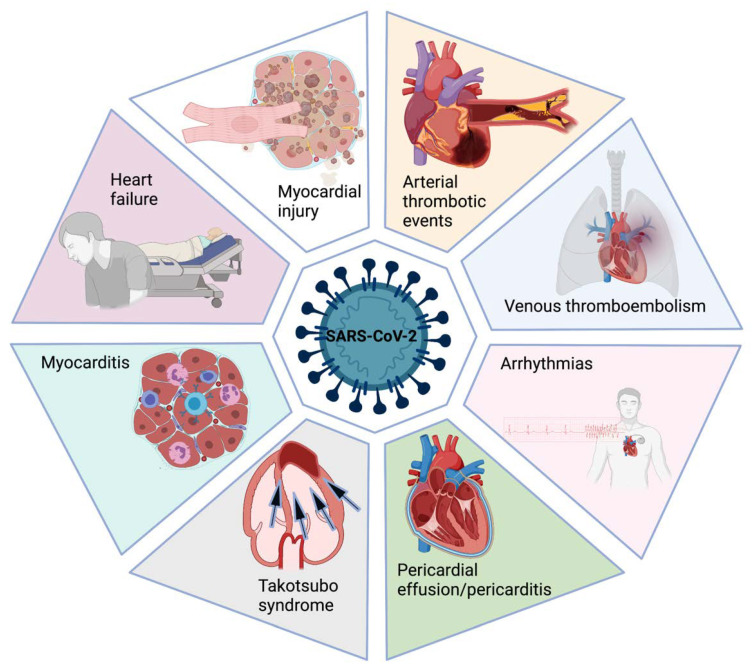
COVID-19 cardiovascular manifestations. Cardiovascular involvement due to COVID-19 ranges from asymptomatic myocardial injury to more severe complications such as type 1 myocardial infarction, arterial or venous thromboembolism, or myocarditis.

Currently, there is a cumulative of data about the cardiovascular effects of COVID-19 during the acute phase of the disease. Meanwhile, information about the post-acute and chronic phases is still insufficient. Moreover, it is unknown if cardiac involvement during the acute phase of COVID-19 may have a clinical impact on the long-term prognosis, including worsening previous cardiac disease or developing new cardiac conditions (Figure 1).

This review aims to summarize the current evidence about the acute and chronic cardiovascular impact of COVID-19. It will describe the plausible pathophysiologic mechanism implicated, acute cardiac manifestations, cardiac imaging findings, and the evidence about the post-acute and chronic effects of COVID-19 on the cardiovascular system.

## 2. Link between SARS-CoV-2, ACE2, and the Cardiovascular System

The renin–angiotensin–aldosterone system (RAAS) has a role not only as a cardiovascular circulating hormonal system but also as a local system that works synergistically with the circulating. The RAAS locally generates signals to regulate different functions that contribute to the homeostasis or damage in the local tissues [7,8]. There are two RAAS pathways: the classic RAAS pathway, which is mediated by the angiotensin II (AII), and the non-classic pathway, which is mediated by the angiotensin 1–7 (A1-7). Both pathways are present in the lungs, the myocardium, and vascular tissues [7]. In the myocardium, a homeostatic balance exists between the classic and non-classic pathways. The classic RAAS activity increase with a non-classic pathway decrease is associated with deleterious cardiovascular effects. Of note, some of the consequences of this imbalanced activity are cardiac hypertrophy, fibrosis, and dysfunction leading to heart failure (HF) and atrial fibrillation (AF) [9].

The relationship of the RAAS with the SARS-CoV-2 was exposed when the angiotensin-converting enzyme 2 (ACE2) was postulated as a potential local receptor for the spike (S) protein of the virus, with a key role in the viral entrance at a cellular level [10]. The ACE2 exists in two forms, a soluble form and a transmembrane protein. The transmembrane ACE2 is present in the alveolar epithelial cells, where the SARS-CoV-2 binds through the S protein to enter the pneumocyte. However, to complete this process, the TMPRSS-2 that primes the S protein is also needed for facilitating the fusion to the cellular membrane and viral entrance [11].

**Figure 2:**
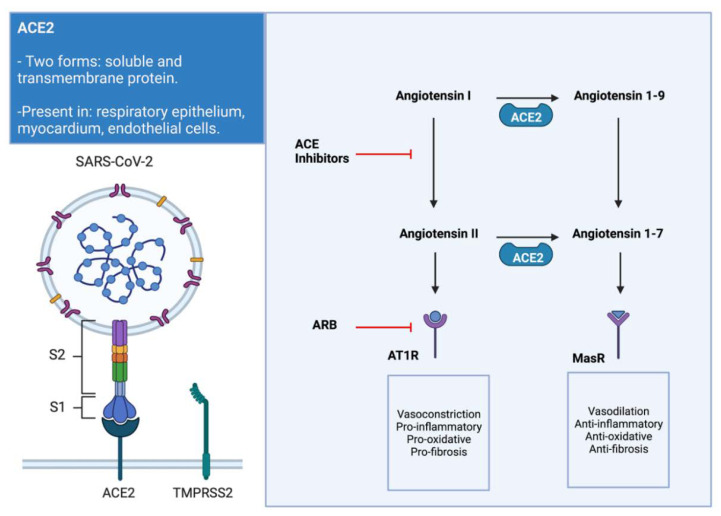
Relationship of angiotensin-converting enzyme 2 (ACE2) with SARS-CoV-2. SARS-CoV-2 binds to the cells through the transmembrane ACE2, which is present in several tissues. After the union to the receptor, the entrance to the cell is facilitated by another transmembrane protein (TPMPRSS2). Furthermore, the possible downregulation of the ACE2 activity with decreased conversion of angiotensin II to angiotensin 1–7 leads to detrimental effects in the myocardium and other systems. Sustained stimulation of the angiotensin receptors (AT1R) by angiotensin II and decreased stimulation of the angiotensin 1–7 receptor (MasR) is associated with adverse myocardial remodeling for predominance of profibrotic and pro-oxidative mechanisms. In addition, the sites of action of the ACE inhibitors and angiotensin receptor blockers (ARB) can be seen in the figure. These two drugs help to reduce the deleterious effects of the RAAS classic pathway overstimulation.

When SARS-CoV-2 binds to ACE2 at the myocardium and endothelial cells, it produces a downregulation in the ACE2 activity, reducing the conversion of AII to A1-7. This decreased activity produces an enhancement of the classic pathway of the RAAS, with subsequent deleterious consequences. The predominance of the classic RAAS pathway led by the AII activity is related to vasoconstriction, fibrosis, pro-inflammatory effects, and reduced collagenase enzymes in the heart. These changes in the RAAS pathways can posteriorly lead to adverse remodeling of the myocardial tissue of the atriums and ventricles (Figure 2) [10,12].

Early in the pandemic, it was thought that the RAAS blockers such as the angiotensin-converting enzyme inhibitors (ACEI) and the angiotensin receptor blockers (ARB) could contribute to a worse evolution of COVID-19 patients. This hypothesis was drawn because these drugs increase the expression of the ACE2 messenger ribonucleic acid (mRNA), boosting the number of potential receptors for the virus [13]. However, this was not confirmed in posterior studies, and there is no established relationship between the ACE2 activity and the COVID-19 associated mortality [8,14]. Furthermore, ACEIs and ARBs could be protective due to the inhibition of the harmful effects of the classic RAAS pathway, and the discontinuation of these drugs could lead to worse outcomes in the patients on chronic treatment [8].

Observational studies have shown no difference in the death rate between the use of RAAS blockers (ACEIs and ARBs) and other antihypertensive drugs (non-RAAS blockers) in COVID-19 patients [15,16]. One retrospective observational study has even shown that the use of ACEI or ARBs are associated with reduced mortality in hospitalized COVID-19 patients who received treatment with these antihypertensives [17]. However, in the randomized clinical trial BRACE CORONA, there was no significant difference in death, cardiovascular outcomes, and between continuing vs. discontinuing ACEI/ARBs in patients with mild to moderate COVID-19 who took these drugs previous to hospital admission [18]. Nevertheless, the BRACE CORONA trial was criticized because of the low mortality and the small proportion of patients using ACEIs and ARBs due to heart failure. Therefore, we will probably need more trials to understand the best approach for these patients. Several clinical trials are ongoing to answer these questions (ClinicalTrials.gov identifier, NCT04351581, NCT04353596, NCT04329195, NCT04351724). In the meantime, most scientific societies suggest continuing ACEIs and ARBs in patients who were receiving treatment with these drugs previously [19,20].

## 3. Myocardial Injury during Acute Disease

Since early 2020, the first data of the hospitalized patients in Wuhan showed that severe COVID-19 was associated with a higher incidence of myocardial injury. Myocardial injury was defined as blood levels of cardiac biomarkers (high sensitivity troponin I or T, in most cases) above the 99th percentile upper reference limit, regardless of new abnormalities in electrocardiography and echocardiography. The significant rise of cardiac biomarkers in blood was an independent predictor of worse in-hospital prognosis and mortality [21,22]. The incidence of myocardial injury varies between studies, depending on the sample and characteristics of the included patients. A review of 26 studies including 11,685 patients showed a weighted pooled prevalence of myocardial injury of 20% (ranging from 5% to 38% depending on the criteria used in the analysis) [23].

However, the exact mechanisms that conduce to cardiac injury development are not entirely understood. The most accepted potential mechanism will be discussed below.

### 3.1. Direct Tissue Injury and Myocarditis

As we previously mentioned, the myocardium and endothelial cells have an important expression of the transmembrane ACE2, representing a probable link for the direct viral invasion. It was hypothesized that the presence of this enzyme in these tissues could contribute to the development of myocarditis [24]. However, large histopathologic studies have demonstrated that the prevalence of myocarditis due to direct viral invasion and toxicity to the cardiomyocytes is probably rare. Furthermore, none of these groups of myocarditis diagnosis meet the Dallas criteria for myocarditis [25,26]. Of note, even when viral RNA was isolated in the cardiac tissue, in situ hybridization localizes the infection more in the interstitial tissue and infiltrating macrophages [25]. However, cardiac imaging found signs of myocarditis even in 30% of the evaluated COVID-19 patients, which is probably not related to a direct invasion but to an indirect inflammatory state [27].

### 3.2. Systemic Hyperinflammatory Response and Critical Illness

In COVID-19 patients, the systemic hyperinflammatory state plays a central role in the pathophysiological development of this disease. This inflammatory environment led by a cytokine storm with interleukin 1 (IL-1), interleukin 6 (IL-6), and tumoral necrosis factor (TNF) could conduce to the rupture of previous stable atherosclerotic plaques or accelerate the atherogenic process. The cytokines probably act by recruiting inflammatory cells through increased expression of adhesion molecules [28,29]. Observational studies have reported that elevated inflammatory biomarkers such as C-reactive protein (CRP), IL-6, and ferritin were related to higher incidences of myocardial injury in COVID-19 patients [30,31]. Furthermore, cardiomyocytes express receptors for the TNF and IL-6, and the binding of these molecules leads to a decreased inotropic response secondary to catecholamine signaling alteration and cytotoxic lesion [32].

Metkus et al. have compared troponin elevation in COVID-19 patients with acute respiratory distress syndrome (ARDS) versus non-COVID-19 patients with ARDS and demonstrated that myocardial injury was less common in COVID-19 patients after accounting for the degree of severity and organ dysfunction [33]. Moreover, it is known that other critical patients, such as those with bacterial sepsis, can show high values of troponin in blood [34]. This highlights that critical illness itself can produce cardiac biomarker elevation in blood.

Critical illness with increased metabolic oxygen requirements and decreased oxygen supply can provoke a mismatch in the demand/supply balance with type 2 myocardial infarction development [35]. This imbalance is probably the more accepted mechanism for troponin elevation in the absence of other cardiovascular manifestations (chest pain, electrocardiogram ischemic alterations, wall motion abnormalities in echocardiogram) during the acute phase of COVID-19 [36].

### 3.3. Macro and Microvascular Thrombi

The severe inflammatory environment in COVID-19 is also closely related to the development of coagulopathy and a resultant pro-thrombotic state [2]. The activation of thrombosis in this context has many pathways, including a higher expression of tissue factor in response to IL-6, excessive complement activation, or neutrophil extracellular traps (NETs). All the coagulation disturbances were described under the denomination of COVID-19 associated coagulopathy (CAC). The severity of the CAC is related to worse in-hospital outcomes [37,38].

The development of endotheliitis has been reported as a contributing cause of thrombotic episodes. Several histological studies have shown an essential component of lymphocytic infiltration with apoptotic bodies in the endothelium. The significant presence of ACE2 in the endothelial cells may explain this phenomenon [39,40].

Thrombotic complications associated with COVID-19 severe disease have been described since the first months of 2020. The localization of the thrombi affects all the vascular territories, from venous to arteries and microcirculation [41,42].

## 4. Cardiovascular Specific Complications

In addition to the myocardial injury itself, different cardiovascular complications have been reported during acute COVID-19. It is important to remember that myocardial injury was defined as an elevation of cardiac biomarkers regardless of new abnormalities in electrocardiography and echocardiography [21]. The absence of more extensive cardiovascular imaging techniques and other explorations in these patients may imply that some patients had misdiagnosed some specific entities. Of note, myocardial injury is not the equivalent of myocardial infarction or myocarditis. Here, we detail some of the potential mechanisms of cardiac involvement.

### 4.1. Acute Coronary Syndromes

SARS-CoV-2 infection has been considered a potential triggering factor for acute coronary syndromes (ACS). Plaque rupture may be triggered by the concomitant macrophage hyperactivation, collagenases secretion, and subsequent degradation of the atheromatic fibrous cap [43].

On the other hand, hypoxemia secondary to ARDS and higher metabolic requirements associated with the hyperinflammatory state could favor an imbalance in myocardial oxygen supply/demand and lead to type 2 myocardial infarction. Vasospasm and coronary artery dissection have also been described in the pathogenesis of ACS in COVID-19 [44,45].

Despite the possible association between ACS and COVID-19, a reduction in the rate of percutaneous coronary interventions (PCI) has been reported in different countries, including the United States, Italy, and Spain (38, 32, and 40%, respectively) during the peak of the pandemic [46]. Of note, observational data suggested an increase in out-of-hospital cardiac arrest during the pandemic’s beginning, suggesting that the decrease in PCI could be associated with an increase in sudden death [47].

### 4.2. Myocarditis

Myocarditis was suggested as one of the possible mechanisms of myocardial injury. As we mentioned, a direct invasion by binding to ACE2 present in myocardial tissues has been proposed [11,12]. Nonetheless, the current evidence cannot rule out this possibility. Other mechanisms of injury appear to be more likely [25]. Inflammatory mononuclear infiltrate in hearts from autopsies was recognized but without evidence of direct virus replication in the myocardium. This evidence suggests that myocarditis may be caused by indirect damage [48]. Furthermore, Kawakami et al. analyzed the samples of 16 patients who died due to COVID-19. None of them met myocarditis criteria, and SARS-CoV-2 RNA was found in the atrium of only two individuals (not in ventricles), despite all of them presenting RNA of the virus in the lungs [49].

The incidence ranges from 4.5 to 30% [27,49]. These percentages come from histological studies performed in most cases in highly selected cases, and the actual frequency of myocarditis in COVID-19 patients is probably much lower. However, the true incidence of myocarditis is unknown and varies depending on the diagnostic criteria and the population included in the studies.

Up until now, considering the available data, endomyocardial biopsy (EMB) is not recommended to be routinely used in suspected cases of COVID-19-related myocarditis [49]. EMB has poor diagnostic performance due to the low incidence, high false-negatives rate, and unclear therapeutic implications. Moreover, non-invasive tests such as CMR may provide high-quality information for myocarditis diagnosis. Eventually, EMB could be used only in worst-case scenarios, such as fulminant heart failure, mainly to rule out other potential diagnoses.

### 4.3. Heart Failure

Heart failure was a common finding in acute COVID-19 patients. First, it could be a consequence of decompensation of a previous chronic heart failure. The different mechanisms can contribute, including hyperinflammation, coagulation abnormalities, and increased metabolic requirements [50].

Second, heart failure can appear in previously healthy patients. Cardiac involvement in COVID-19 patients with decreased left ventricle (LV) ejection fraction or due to diastolic dysfunction has been reported [50]. The same mechanisms that would produce myocardial injury and myocarditis have been implicated.

### 4.4. Takotsubo Syndrome

The current pandemic context may represent a potential trigger for Takotsubo syndrome due to severe respiratory distress and the deep emotional stress caused by isolation that can lead to an excessive release of catecholamines. So far, there are very heterogeneous case reports showing laboratory and electrocardiogram (ECG) changes typical of myocardial ischemia such as increased cardiac biomarkers in blood, T-wave inversion, and ST-segment elevation, in addition to transthoracic echocardiography showing LV dysfunction, associated with apical ballooning, characteristic of Takotsubo syndrome [51].

### 4.5. Venous Thromboembolic Events

Among COVID-19 patients, the incidence of venous thromboembolism (VTE, composed of deep venous thrombosis [DVT] and pulmonary embolism [PE]), is more common in critically ill patients. Factors contributing to VTE are immobilization, hyperinflammation, hypoxia, and endothelial cell injury [52].

Wang et al. found that 40% of COVID-19 patients are at high risk of DVP and PE. [43]. The prevalence of PE in patients with severe disease ranged from 17 to 47%, and many of the thromboembolic events may occur despite thromboprophylaxis [53,54]. However, the exact prevalence of VTE is challenging to determine. Of note, prophylactic therapeutic anticoagulation regimen dosing is associated with a lower incidence of VTE, specifically PE, compared to conventional prophylactic dosing. There has been no clear benefit in terms of mortality reduction, and an increase in bleeding has been observed in patients treated with prophylactic therapeutic anticoagulation [55].

### 4.6. Arrhythmias

The risk factors for arrhythmias during acute COVID-19 are the presence of myocardial injury or ischemia, hypoxia, shock, and electrolyte disturbances. Moreover, it is important to note the risk of arrhythmias in patients who received medications that prolong the QT interval during the beginning of the pandemic [56].

The most frequent arrhythmias in an observational study were AF (3.6%), non-sustained ventricular tachycardia (1.4%), cardiac arrest (1.3%), and bradyarrhythmias (1.3%) [57].

## 5. Cardiac Imaging Findings

**Figure 3:**
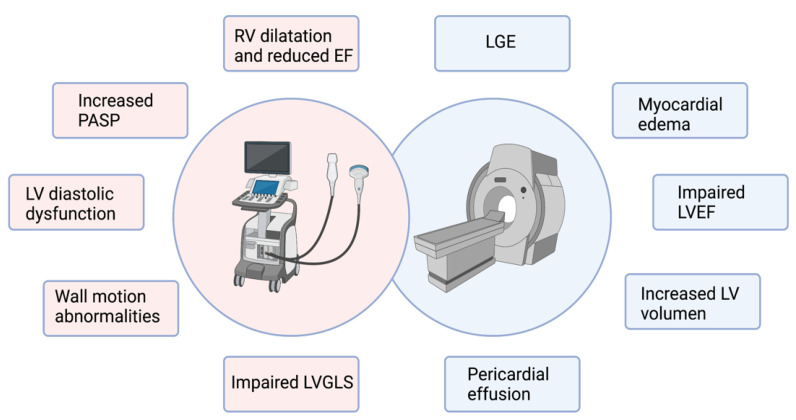
Cardiac imaging techniques main findings in COVID-19 patients with cardiac involvement. On the left, echocardiography findings are summarized. On the right, CMR findings. CMR, cardiac magnetic resonance; LGE, late gadolinium enhancement; LV, left ventricle; LVGLS, left ventricle global longitudinal strain; PASP, pulmonary artery systolic pressure; RV, right ventricle; TAPSE, anterior tricuspid plane systolic excursion.

Since the physicians have obtained data about cardiac involvement during the COVID-19 acute phase, several studies with cardiac imaging have been carried out to determine different markers to guide the decision-making process and understand this disease’s cardiovascular evolution. Echocardiography and cardiac magnetic resonance (CMR) were the most explored techniques [58,59]. The main findings of both imaging techniques are summarized in Figure 3.

### 5.1. Echocardiography

An important number of reports describing echocardiographic findings have been published. However, the prognostic role of these explorations needs to be defined due to the heterogeneous findings. Some reasons that may explain these mixed results are the patient bias selection, difference in the disease severity, and difference among local protocols of imaging assessment [60,61].

One of the main findings in echocardiographic studies is the right ventricle (RV) impairment. The pathophysiology of COVID-19 with an important incidence of ARDS can lead to the development of RV dysfunction due to the increased pulmonary microvascular resistance, accompanied by microvascular thrombi, high rate of PE, and even due to mechanical ventilation itself [58,59]. Manzur-Sandoval et al. found a high frequency of echocardiographic signs of RV dysfunction (reduced fractional shortening, reduced tricuspid annular plane systolic excursion, and RV dilatation) in patients who required hospital admission. The presence of RV dysfunction and elevated pulmonary artery systolic pressure (PASP) were associated with higher in-hospital mortality [62]. In line with these findings, another observational study focused on intensive care unit (ICU) patients found higher rates of increased PASP (69%) and RV dilatation (28%) than studies that included non-ICU admitted patients. [63].

Regarding the LV, the findings differed between studies due to the reasons stated previously. One of the frequent observations was the LV’s diastolic dysfunction, which was found in up to 16% of COVID-19 patients [64]. A reduced LV ejection fraction (LVEF) was less common than diastolic dysfunction, with a reported prevalence of 10% [65]. Wall motion abnormalities were reported in 14 to 26% of the COVID-19 patients who underwent echocardiography. The most commonly affected areas were the mid and apical segments [66,67]. Cases with severely reduced LVEF and severe LV dilatation were less common, with punctual cases of acute heart failure and cardiogenic shock reported [68,69].

Giustino et al. compared COVID-19 patients with myocardial injury vs. without myocardial injury to characterize echocardiographic findings. They observed that patients with myocardial injury showed a higher prevalence of wall motion abnormalities, global LV dysfunction, LV diastolic dysfunction grade II or III, right ventricular dysfunction, and pericardial effusions. Myocardial injury plus echocardiographic abnormalities were associated with a worse prognosis [67]. LV global longitudinal strain (LVGLS) was more frequently impaired in patients with myocardial injury than those without [70,71]. Furthermore, LVGLS was more prevalent in patients with even mild COVID-19 than healthy controls [72,73].

### 5.2. Cardiac Magnetic Resonance

The interest in CMR assessment of COVID-19 patients is related to the myocarditis reports. However, due to prolonged acquisition time and high contagiousness of COVID-19 infection, CMR has been used only in sporadic opportunities during the acute phase of the disease. Most of the data have been obtained in post-acute COVID-19 or later. Reports of the incidence of cardiac involvement and abnormalities shown with CMR have been heterogeneous among studies [74,75].

Esposito et al. reported a series of 10 patients with suspected myocarditis who underwent CMR, which was performed within one week from troponin rise and the onset of cardiac symptoms. Eight patients presented myocardial edema and met the Lake Louise criteria for myocarditis diagnosis [76].

A German study including 100 patients who recently recovered from COVID-19 illness observed that 78% of them presented cardiac involvement in the CMR. An important characteristic of this study is that 67% had mild disease. Compared with healthy controls and risk-factor matched controls, COVID-19 recovered patients showed lower LVEF and higher LV volume. Patients with cardiac involvement showed a raised myocardial native T1 and T2, myocardial late gadolinium enhancement (LGE), or pericardial enhancement. These findings were recorded at a median of 71 days after COVID-19 diagnosis and demonstrated cardiac abnormalities beyond the acute phase of the disease [77,78].

In another study, 148 hospitalized patients with severe COVID-19 and elevated troponin were evaluated with CMR ~2 months after diagnosis or discharge. The main findings were LGE in 54% of the patients, of whom 26% showed non-ischemic and 22% ischemic patterns. Signs of active myocarditis were present in 30%, and impaired LVEF in 11% of the patients [27].

Currently, there is no clear evidence to suggest the best timing for a CMR in the context of a suspected case of myocarditis. However, in the setting of high suspicion, CMR could be performed within a week of the beginning of the symptoms if the patient remains stable and can tolerate the procedure. Routine CMR in the follow-up may not be recommended. Future data will clarify questions about the best approach in this kind of case [75].

## 6. Cardiac Biomarkers 

The elevation of cardiac injury biomarkers in blood was related to worse in-hospital outcomes in COVID-19 patients. These markers more frequently rose in patients with cardiovascular risk factors and prior cardiovascular disease, including ischemic heart disease, chronic heart failure, peripheral vascular disease, and history of stroke [21,22,31].

Different biomarkers have shown a prognostic value for in-hospital and short-term (until 28 days) mortality (Table 1) [79,80]. Higher venous blood concentrations of creatin kinase isoenzyme-MB (CK-MB), myoglobin, high-sensitivity troponin I, and N-terminal pro-brain natriuretic peptide (NT-proBNP) were found to be associated with COVID-19 severity and case fatality rate. These findings could assist in better management of COVID-19 patients to improve outcomes in the short term. However, the long-term impact of the cardiac affectation during the acute phase and the specific follow-up required in these settings are still unknown.

## 7. Long COVID and Post-Acute COVID-19 Syndrome

With the advance of the pandemic, physicians have noticed that some patients who have recovered from the acute phase of COVID-19 remained with a varied set of symptoms. The clinical presentation includes racing heartbeat, shortness of breath, foggy thinking, persistent loss of sense of smell, and achy joints [84]. 

**Figure 4:**
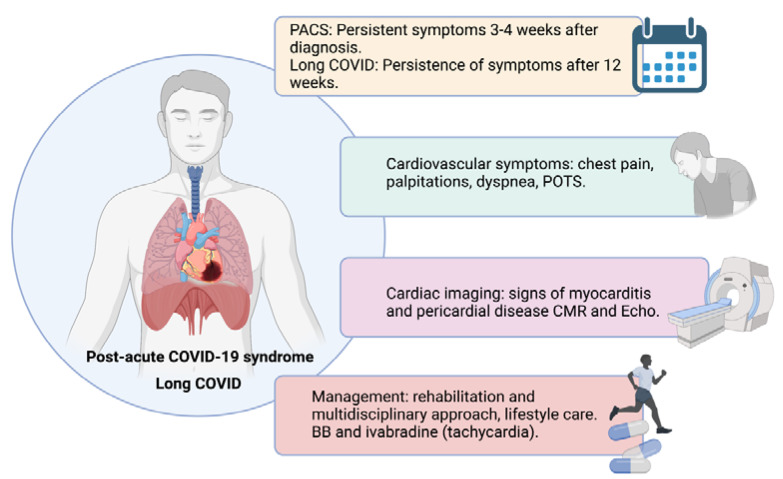
Post-acute COVID-19 syndrome (PACS) and Long COVID key points. BB, beta blockers; CMR, cardiac magnetic resonance; POTS, postural orthostatic tachycardia syndrome.

Even though some of the following terms have been used as synonymous, several definitions have been raised. “Post-acute COVID-19 syndrome” (PACS) was stated for persistent symptoms after three to four weeks [85,86]. “Chronic COVID-19” or “Long COVID” have been used for persistent symptoms for more than 12 weeks or three months. Furthermore, the National Institutes of Health (NIH) has referred to Long COVID as “post-acute sequelae of SARS-CoV-2 infection” [87]. Some of the key points regarding PACS are summarized in Figure 4.

The pathophysiologic mechanisms for these symptoms are not completely understood, and they are still under study, including the cardiac and cardiovascular manifestations [88]. In a follow-up study including 384 hospitalized COVID-19 patients, the D-dimer, CRP, and ferritin levels normalized within two months after discharge. Moreover, chest radiography remained abnormal only in 10% of these patients. However, persistent symptoms (fatigue and breathlessness) were found in more than two-thirds of them despite laboratory and radiographic improvement [89]. This incongruency suggests a different mechanism than those involved in the acute phase.

Available data suggest that Long COVID can be presented even in patients with the mild disease during the acute phase [88,90]., even though the frequency of persistence of symptoms is higher in patients who developed and recovered from ARDS. Taboada et al. reported that in 91 survivors from COVID-19 related ARDS, 67% showed a decreased quality of life and 63% had a lowered functional status at six-month follow-up. The need for mechanical ventilation, length of ICU stay, and length of hospital stay were associated with worse quality of life and functional status [91]. ARDS could be related to persistent radiological and functional lung impairment, but an eventual relation with worse cardiovascular outcomes remains unclear [92].

Regarding cardiovascular symptoms, the myocardial injury could be the triggering factor of an inflammatory cascade and subsequent fibrosis. Furthermore, the extent and distribution of this inflammatory reaction could lead to adverse ventricular remodeling and malignant arrhythmias [93]. The possible role of the inflammatory mechanisms in the pathophysiology of Long COVID is still under study, and several hypotheses were developed. Elevated levels of pro-inflammatory markers (e.g., CRP, IL-6, and D-dimer) and lymphopenia have been associated with prolonged COVID [90]. A recent case-control study found elevated levels of vessel-related pro-inflammatory biomarkers, which were correlated with lung damage among COVID-19 patients discharged three months earlier [94]. These reports suggest that unresolved inflammation may explain part of the pathophysiology of Long COVID. However, more data is needed to determine the mechanisms and the potential therapeutic measures to normalize patients’ long-term prognosis.

Another potential mechanism can be the persistent dysregulation of the RAAS with the mechanisms that we have stated previously [9,10,11].

Cardiovascular manifestations in the post-acute phase of COVID-19 described in the studies so far have ranged from asymptomatic changes in CMR exploration to symptoms of heart failure, including palpitations, tightness, dizziness, and chest pain. The frequency of them varied greatly between different reports [95,96,97]. Most of these studies were observational and without a control arm in some cases. Furthermore, reports to date have included various patients with diverse baseline profiles, demographic characteristics, and illness severity.

In addition to the mentioned manifestations, it has gained notoriety from a specific syndrome that has been called “postural orthostatic tachycardia syndrome” (POTS) [98]. POTS is defined as an inappropriate rise in heart rate without change in blood pressure upon movement from a recumbent to an upright position [99]. The pathophysiology is unknown, but it may be associated with post-viral illness [100]. Some hypotheses attribute this syndrome to the interaction between SARS-CoV-2 and ACE2 presented on the neurons, leading to hypotension and dysautonomia. Of note, POTS has been proposed as a possible cause of palpitations, chest pain, and dizziness in patients with Long COVID [101]. There are several reports of cases and case series after COVID-19 infection with the mentioned symptoms, in which diagnosis was established through tilt table test (TTT) [98,102,103]. In the larger case series, 20 patients with suspected POTS were evaluated, 70% of them were female, and 80% needed pharmacological treatment. Finally, POTS were confirmed in 15 of them [103]. 

The better treatment for PACS remains unknown. It has been suggested that early rehabilitation and a multidisciplinary approach must be the cornerstone [104]. Regardless of cardiac symptoms, lifestyle care (adequate hydration and salt balance) and the use of beta-blockers or ivabradine for tachycardia could be useful in this setting [99].

Although the symptoms of the post-acute phase of the disease have been in focus, cardiac imaging follow-up of COVID-19-recovered patients raised concerns from the medical community. In a study performed in Ohio, 26 athletes with asymptomatic or mildly symptomatic previous COVID-19 were evaluated with CMR; four of them met two main features of the updated Lake Louise criteria for myocarditis, including myocardial edema and non-ischemic LGE [105].

Similar observations were not found in other explorations performed in athletes. A study including 789 professional athletes recovered from COVID-19 who were tested before going back to competition found evidence of myocarditis only in five athletes (0.6%) [106]. Another big study with 3018 athletes who underwent cardiac evaluation (electrocardiogram, echocardiography, and CMR) found low incidence of cardiac involvement (0.7%) [107].

## 8. Chronic Effects of COVID-19 on the Cardiovascular System: Evidence at Mid- and Long-Term Follow-Up

The data about the sequelae of COVID-19 on the cardiovascular system is increasing. However, there are still many questions regarding the pathophysiologic mechanisms and the real implications of imaging findings of the acute and post-acute phase on the long-term prognosis. Studies suggested that patients who have recovered from COVID-19 will present a greater risk for a range of cardiac diseases, including heart failure, myocardial infarction (MI), and arrhythmia in the mid- and long-term [95].

Concerning the mid-term follow-up (about 3 to 6 months), Sonnweber et al. followed 145 patients who recovered from COVID-19 until a mean of 103 ± 21 days and found that 41% exhibited persisted symptoms (36% dyspnea). Echocardiography showed diastolic dysfunction (55%) and signs of pulmonary hypertension (10%) as the most common findings [108].

Li et al. performed a study with 40 patients who recovered from COVID-19 moderate or severe pneumonia and had no cardiovascular medical history. They were evaluated with CMR at a median of 124 ± 17 days after discharge compared with control subjects. LGE was present only in one patient (3%). However, those with previous COVID-19 pneumonia showed higher median extracellular volume (ECV) and worse median two-dimensional LV global longitudinal strain [109]. 

In opposition, in a case-control study with a 6-month follow-up, 74 asymptomatic or mildly symptomatic seropositive healthcare workers were compared with 75 seronegative controls. There were no differences in cardiac structure, LVEF, or cardiac biomarkers between both groups [110].

Regarding clinical outcomes, in a study with 620 COVID-19 patients and 245 controls, despite a higher risk of death and CV complications during the index hospitalization, there were no differences in the major adverse cardiac events at 6-month follow-up. [102]. Conversely, a UK study with a mean follow-up of 140 days compared 47,780 COVID-19 survivors with matched controls from the general population. The authors found that COVID-19 re-covered patients presented a higher risk of hospital readmission, all-cause death, and major adverse cardiovascular events [111].

Results of more extended follow-up studies are becoming available. In a 12-month follow-up study assessing Long COVID symptoms in 96 patients (32.3% with previous admission during the acute phase), it was found that only 22.9% of them were free of symptoms. Reduced exercise capacity (56.3%), fatigue (53.1%), and dyspnea (37.5%) were among the most common referred symptoms [112]. In another prospective study, Maestrini et al. found that at one-year follow-up of 118 COVID-19 survivors, 47.5% presented persistent symptoms, and fatigue (14.2%) was the most frequent. Other symptoms of interest for our revision were dyspnea 10.8%, palpitations 4.2%, and chest pain 1.7%. In addition, 11 patients (9.3%) required new hospital admission [113].

Available data suggest that persistent cardiovascular symptoms can be expected in a non-negligible proportion of COVID-19 survivors even in the long term. Several studies are currently being conducted to find out the long-term implications of COVID-19. One of them, the CV-COVID-19 registry (NCT04359927), aims to determine the frequency of clinically relevant endpoints such as cardiovascular mortality, ACS, stroke, PE, and hospital admission due to HF [114]. Other studies with similar aims are NCT04606732, NCT04701515, and NCT04605965. Future research perspectives are shown in Table 2.

## 9. COVID-19 Vaccine Side Effects

The emergence of the COVID-19 vaccines represents the most effective pharmacological intervention for preventing infection or severe illness [115]. Several vaccine platforms were developed, and some others are currently in the process of development [116]. mRNA-based vaccines were the first to be applied and have shown acceptable safety and promising efficacy. Other platforms have used an adenovirus modified with the addition of the S protein.

Although the vaccines have reported safety, several types of adverse events have been reported. These range from mild symptoms to some more specific organ effects. Thrombosis has emerged as a major concern in patients who received adenovirus-based vaccines. Some cases of cerebral venous sinus thrombosis (CVST), especially in women, were communicated. However, Bikdeli et al. evaluated the incidence of this complication in patients vaccinated against COVID-19 and compared it with the general population and COVID-19 patients. They found that CVST was rare among people who received an adenovirus-based vaccine and general population but appeared to be several-fold more frequent in COVID-19 hospitalized patients [117].

On the other hand, Montgomery et al. reported 23 cases of probable myocarditis among military males who received an mRNA-based vaccine against COVID-19. A total of eight of them underwent CMR, confirming the diagnosis in all cases. The mean time to the onset of the symptoms was 50 h. All the patients recovered posteriorly. These episodes occurred against the backdrop of 2.8 million doses of mRNA COVID-19 vaccines ad-ministered, so it is considered a rare event [118]. On the other hand, information about vaccine-related adverse events in children and adolescents is still scarce. Marshall et al. reported seven cases of adolescents who developed myocarditis within four days following mRNA vaccine second dose application. All of them showed troponin I elevation and LGE in the CMR, with a complete recovery [119]. In the same way, Jain et al. reported that myocarditis after mRNA vaccines evolved mainly with mild symptoms in 63 adolescent patients, with the resolution of the clinical manifestations at a mean of 35 days [120]. Additional follow-up will probably be needed to determine the actual incidence and prognosis of vaccine-related myocarditis in the general population.

## 10. Conclusions

Since the beginning of the COVID-19 pandemic, the cardiovascular system had been closely linked to this disease. The acute cardiovascular involvement, including myocardial injury, ACS, myocarditis, and PE, is related to worse short-term outcomes and mortality. Several mechanisms are probably implicated. However, cardiac damage seems to be multi-factorial, with a key role in impaired regulation of RAAS, inflammation, and coagulopathy disorders.

Imaging explorations, specially CMR, have provided accurate information about structural and functional changes in the acute and post-acute phases. Moreover, this diagnostic tool will be fundamental in long-term assessments of cardiac involvement.

With the evolution of the pandemic, COVID-19 survivors will have to face new challenges, such as chronic sequelae and persistent symptoms that are part of Long COVID. This last syndrome will open a new window in biomedical research with several questions to be answered.

## Figures and Tables

**Table 1:** Main cardiac biomarkers used in clinical practice in patients with COVID-19.

Cardiac Biomarker	Clinical Utility	Pathophysiological Processes Involved in COVID-19	Available Evidence	Comment
Cardiac Troponin I or T	Quantitative marker of cardiomyocyte injury.	Chronic elevation: -Pre-existing cardiac condition. Acute non-ischemic elevation: -Direct effect of SARS-CoV-2 on the myocardial cells. -Myocarditis. -Takotsubo syndrome. -Pulmonary embolism. Acute ischemic elevation: -Type 1 MI. -Type 2 MI (shock, hypoxia, or tachycardia).	-Strong and consistent association with in-hospital and 28-day mortality [81]. -Systematic testing is a matter of debate [82]. -No evidence of association in the decision-making process among testing, triggered intervention, and improvement in clinical outcomes [81].	If the myocardial injury is associated with clinical ischemic symptoms (chest pain, ischemic ECG changes including Q waves, imaging evidence of new loss of viable myocardium, or new regional wall motion motility abnormality), invasive management could be appropriate according to the clinical condition of the patient [2].
BNP, NT-proBNP, or ANP	Quantitative biomarkers of hemodynamic myocardial stress and heart failure.	ANP: -Atrial volume or pressure increase. BNP or NT-proBNP: -Left ventricular systolic or diastolic dysfunction. -Right ventricle dysfunction (an increase of overload and hypoxemia). -Valvular dysfunction.	-Associated with critical illness and mortality [83]. -Currently recommended cut-offs should not be applied in critically ill patients with ARDS or septic shock [81]. -Systematic testing is a matter of debate [82].	Natriuretic peptides may be useful when heart failure is suspected. In a proper clinical context, the simultaneous measurement of natriuretic peptides and cardiac troponin may be helpful to rule in or rule out acute cardiac involvement.
D-Dimer	Generated by cleavage of fibrin monomers by plasmin, indicating the presence of thrombus formation and subsequent fibrinolysis.	-Blood levels are correlated with the degree and stage of COVID-19–associated hemostatic abnormalities. -In low-risk patients, may be useful for rule out VTE. -In very high concentrations (e.g., >10-times the ULN) having a high positive predictive value for the diagnosis of VTE. -May be used for the diagnosis and monitoring of disseminated intravascular coagulation associated with sepsis or shock.	-Contributes to early risk assessment and may provide guidance to select candidates’ escalated dose anticoagulation [81]. -May help to anticipate potential unresponsiveness to therapies, respiratory failure, ARDS, and death [81].	Several randomized clinical trials have included D-dimer as high-risk criteria for selecting patients who may benefit from escalated dose prophylactic anticoagulation. However, mixed results have a potential benefit in some trials and a null effect in others [55].

ANP, atrial natriuretic peptide; ARDS, acute respiratory distress syndrome; BNP, brain natriuretic peptide; COVID-19, coronavirus disease 2019; MI, myocardial infarction; NT-proBNP, N-terminal pro-brain natriuretic peptide; SARS-CoV-2, severe acute respiratory syndrome coronavirus-2; ULN, upper limit of normal; VTE, venous thrombo-embolism.

**Table 2:** Areas of interest in the investigation of COVID-19 and its relationship with the cardiovascular system.

Areas	Relationship with the Cardiovascular System
Myocardial Injury	- Pathophysiological mechanism that links COVID-19 with myocardial injury.
	- Long-term prognostic value of troponin in COVID-19 patients.
	- Relationship of myocardial injury with new treatments, including immunomodulating therapies.
CMR	- Clinical implications and long-term prognostic value of imaging changes in the acute and post-acute phase of the disease.
	- Prevalence of persistence imaging changes at one-year follow-up and their clinical relevance.
Long COVID	- Prevalence of Long COVID in bigger international studies, with stratification according to severity during the acute phase of the disease.
	- Exploration of different therapeutic approaches in Long COVID.
	- Impact of Long COVID on long-term cardiovascular outcomes, including the incidence of heart failure, acute coronary syndrome, arrhythmias, and stroke.
	- Safety for return to play of professional and amateur athletes.
COVID-19 vaccines	- Impact of the vaccine in the development of Long COVID syndrome. - Incidence of myocarditis related to COVID-19 vaccines.
	- Security of vaccines regarding cardiovascular outcomes.

COVID-19, coronavirus disease 2109; CMR, cardiac magnetic resonance.

## Data Availability

Not applicable.
